# Supplementary material for: Dynamic transcriptomic profiles of zebrafish gills in response to zinc supplementation
Source: BMC Genomics. 2010 Oct 11;11:553. doi: 10.1186/1471-2164-11-553 (PMC3091702; doi:10.1186/1471-2164-11-553)
Supplement: Additional file 2 — Interactive Direct Interaction Network representing the molecular interactions between zinc, copper, iron, calcium and proteins encoded by transcripts changed by zinc supplementation. Mini web-site containing index.html and hyperlinked pages in subdirectory describing a Direct Interaction Network automatically generated based on curated interactions contained within the proprietary PathwayArchitect database. Ovals represent proteins and the circles symbolize metal ions. Objects are coloured by their abundance in zebrafish at the time-point they were significantly different from the control is a scale from -4 fold (dark green) to +4 fold (dark red). Where significant differences were found at more than one time-point, the colour overlay shows expression at the first instance. Dark blue squares denote 'binding', and light blue squares 'expression'; green squares stand for 'regulation', green diamonds for 'metabolism', and green circles for 'promoter binding'. Arrow heads indicate directionality of the interaction where annotated. All nodes and edges can be further interrogated by selecting the relative area of the image. [file 1471-2164-11-553-S2.zip › PathwayArchitect Zn xs DIN/750043.html]

# METABOLISM:

|  |  |
| --- | --- |
| Type | METABOLISM |
| Effect | None |


---

|  |  |
| --- | --- |
| Score | 0 |


---

|  |  |
| --- | --- |
| Reference Count | 6 |


---

|  |  |
| --- | --- |
| Mechanism | Unknown |


---

|  |  |
| --- | --- |
| Reference:0 || Sentence | "Ca2+ stimulated Ins(1,4,5)P3 generation only at concentrations above 1 microM." |
| PMID | 8389758 |
| Year | 1993 |
| Species | Human |
| Journal | J Biol Chem |
| RefScore | 0 |
| Source | PArchNLP |
  |
|  |  |


---

|  |  |
| --- | --- |
 Reference:1 || Sentence | "This slow response, its abrogation in Ca2+-depleted medium, the slow and maintained increase in Ca2+ as measured by Quin-2, and the ability of the Ca2+ ionophore A23187 to stimulate Ins(1,4,5)P3 accumulation all suggest that the increase in Ins(1,4,5)P3 occurs, at least in part, as a result of receptor-mediated Ca2+ influx in mitogen-stimulated T lymphocytes." |
| PMID | 2556109 |
| Year | 1989 |
| Species | Human |
| Journal | Biochem J |
| RefScore | 2 |
| Source | PArchNLP |
  ||  |  |


---

|  |  |
| --- | --- |
 Reference:2 || Sentence | "Inhibition of inositol 1,4,5-triphosphate (Ins(1,4,5)P3) binding by Ca2+ and Mg2+ remains a controversial issue: the Ca2+ effect may also be explained by Ins(1,4,5)P3 generation due to a Ca(2+)-sensitive phospholipase C, and the Mg2+ effect by Ins(1,4,5)P3 degradation." |
| PMID | 8389758 |
| Year | 1993 |
| Species | Human |
| Journal | J Biol Chem |
| RefScore | 2 |
| Source | PArchNLP |
  ||  |  |


---

|  |  |
| --- | --- |
 Reference:3 || Sentence | "Thus, we conclude that both Ca2+ and Mg2+ can inhibit Ins(1,4,5)P3 binding independently of phospholipase C activation and Ins(1,4,5)P3 breakdown." |
| PMID | 8389758 |
| Year | 1993 |
| Species | Human |
| Journal | J Biol Chem |
| RefScore | 1 |
| Source | PArchNLP |
  ||  |  |


---

|  |  |
| --- | --- |
 Reference:4 || Sentence | "These findings show, that cultured HCASMC express G protein-coupled purinoceptors, which upon stimulation activate PLC to induce enhanced Ins(1, 4, 5)P3 production causing release of Ca2+ from internal stores." |
| PMID | 8842427 |
| Year | 1996 |
| Species | Human |
| Journal | Br J Pharmacol |
| RefScore | 1 |
| Source | PArchNLP |
  ||  |  |


---

|  |  |
| --- | --- |
 Reference:5 || Sentence | "Entry of Ca2+ into the cells is an important factor, which affects agonist-stimulated accumulation of Ins(1,4,5)P3, as well as absolute values of Li(+)-dependent increase in Ins(1,4,5)P3; however, it is not essential for the manifestation of Li+ effects." |
| PMID | 8869750 |
| Year | 1996 |
| Species | Human |
| Journal | Adv Enzyme Regul |
| RefScore | 1 |
| Source | PArchNLP |
  |


---

|  |  |
| --- | --- |
